# Linkage of Whole Genome Sequencing, Epidemiological, and Clinical Data to Understand the Genetic Diversity and Clinical Outcomes of Shigella flexneri among Men Who Have Sex with Men in England

**DOI:** 10.1128/Spectrum.01213-21

**Published:** 2021-12-15

**Authors:** Holly D. Mitchell, Nicholas R. Thomson, Claire Jenkins, Timothy J. Dallman, Anaïs Painset, Peter Kirwan, Valerie Delpech, Amy F. W. Mikhail, Nigel Field, Gwenda Hughes

**Affiliations:** a Centre for Molecular Epidemiology and Translational Research, Institute for Global Health, University College London, London, UK; b The National Institute for Health Research Health Protection Research Unit (NIHR HPRU) in Blood Borne and Sexually Transmitted Infections at University College London, London, UK; c Parasites and Microbes, Wellcome Trust Sanger Institute, Hinxton, UK; d Department of Pathogen Molecular Biology, London School of Hygiene and Tropical Medicine, London, UK; e National Infection Service, Public Health England, London, UK; f The National Institute for Health Research Health Protection Research Unit (NIHR HPRU) in Gastrointestinal Infections at University of Liverpool, Liverpool, UK; The National University of Singapore and the Genome Institute of Singapore

**Keywords:** whole genome sequencing, surveillance, England, *Shigella*, men who have sex with men

## Abstract

The public health value of whole genome sequencing (WGS) for *Shigella* spp. in England has been limited by a lack of information on sexual identity and behavior. We combined WGS data with other data sources to better understand Shigella flexneri transmission in men who have sex with men (MSM). WGS data for all S. flexneri isolates referred to the national reference laboratory were linked to i) clinical and behavioral data collected in seven of 21 health regions in England using a standardized exposure questionnaire and, ii) national HIV surveillance data. We included 926 S. flexneri isolates, of which 43.0% (*n* = 398) fell phylogenetically within two domestically circulating clades associated with genotypic markers of azithromycin resistance. Approximately one third of isolates in these clades were from people living with HIV, primarily acquired through sex between men. 182 (19.7%) isolates had linked questionnaire data; 88% (84/95) of MSM isolates fell phylogenetically within the domestically circulating clades, while 92% (72/78) of isolates from other cases fell within lineages linked with travel to high-risk regions. There was no evidence of sustained transmission between networks of MSM and the wider community. MSM were more likely to be admitted to hospital and receive antimicrobials. Our study emphasizes the importance of sex between men as a major route of transmission for S. flexneri. Combined WGS, epidemiological and clinical data provide unique insights that can inform contact tracing, clinical management and the delivery of targeted prevention activities. Future studies should investigate why MSM experience more severe clinical outcomes.

**IMPORTANCE** Within the last 2 decades there have been an increasing number of *Shigella* spp. outbreaks among men who have sex with men (MSM) worldwide. In 2015, Public Health England (PHE) introduced routine whole genome sequencing (WGS) for the national surveillance of *Shigella* spp. However, the lack of information on sexual identity and behavior has hindered interpretation. Our study illustrates the power of linking WGS data with epidemiological, behavioral, and clinical data. We provide unique population-level insights into different transmission networks that can inform the delivery of appropriate public health interventions and patient management. Furthermore, we describe and compare clinical characteristics and outcomes of S. flexneri infection in MSM and other exposure groups. We found that MSM were more likely to be admitted to hospital and receive antimicrobials, indicating that their infections were potentially more severe. The exact reasons for this are unclear and require further exploration.

## INTRODUCTION

*Shigella* spp. (Shigella flexneri, Shigella dysenteriae, Shigella sonnei, *and* Shigella boydii) are Gram-negative bacteria and the most common cause of severe dysentery globally (1, 2). Transmission occurs via the fecal-oral route through direct contact with an infected person, or exposure to contaminated surfaces, food, or water. In high-income countries, cases are often linked with travel to regions with poor food and water hygiene, primarily South Asia or sub-Saharan Africa. Sexual transmission can also occur through direct oral-anal contact or through oral sex after sex, via fingers or fomites (3). Within the last 2 decades, S. flexneri and S. sonnei outbreaks among men who have sex with men (MSM) have become more frequent globally, often associated with antimicrobial resistance (4–9).

Shigellosis cases in England have historically been associated with travel to high-risk regions or person-to-person transmission in household or childcare settings (10, 11). However, since 2009, there have been successive increases of S. flexneri (serotypes 2a and 3a) and S. sonnei in adult men reporting no recent foreign travel, while cases in adult men reporting foreign travel, and in women, have remained relatively stable (4, 12, 13). While suggestive of sexual transmission in MSM, the lack of data on sexual identity and behavior has hindered interpretation, with only one small qualitative study in 2012 providing supportive evidence (7). Our previous genomic studies have relied primarily on sex, age, and foreign travel history information to describe *Shigella* spp. sublineages likely being sexually transmitted among MSM (5, 14).

In 2015, to address the lack of evidence for sexual transmission and to inform infection control measures, Public Health England (PHE) piloted a questionnaire to standardize and expand the collection of exposure information on suspected shigellosis cases, including questions on sexual identity and behavior. Concurrently, whole genome sequencing (WGS) was routinely implemented for all *Shigella* spp. isolates referred to the national reference laboratory (15, 16). Previously we showed how these data might be used to distinguish S. flexneri clusters linked through sexual and nonsexual transmission in near real-time (6). Here, we describe the characteristics and genetic diversity of S. flexneri transmission through sex between men, assess any potential overlap with nonsexual transmission, and explore clinical outcomes.

## RESULTS

### Summary of isolates and linked data.

Between August 2015 and July 2017, 926 S. flexneri clonal complex (CC) 245 isolates were referred to the reference laboratory and sequenced. Questionnaire data were available for 182 (19.7%), of which over half (52.2%; *n* = 95) were MSM, 33.5% (*n* = 61) were other adults (heterosexual men or adult women), 9.3% (*n* = 17) were children and 5.0% (*n* = 9) were adult men who did not provide sexual identity or recent behavior information. Data linkage to HIV and AIDS Reporting System (HARS) revealed that 18.7% (173/926) of S. flexneri isolates were taken from people living with HIV (PLWH) (Supplementary File 3).

Isolates with linked questionnaire data represented 38.4% (182/473) of all S. flexneri CC245 isolates referred to the reference laboratory from HPTs participating in the pilot of the questionnaire and 22.8% (182/800) of all isolates referred nationally during the pilot period (August 2015 to March 2017) (Supplementary File 3).

Compared to all isolates included in the study without questionnaire data (*n* = 744), a higher proportion of isolates with linked questionnaire data (*n* = 182) were from cases living in London, adults aged 25–34 years, and cases who had not traveled abroad (Supplementary File 4).

### Genetic diversity.

**FIG 1:**
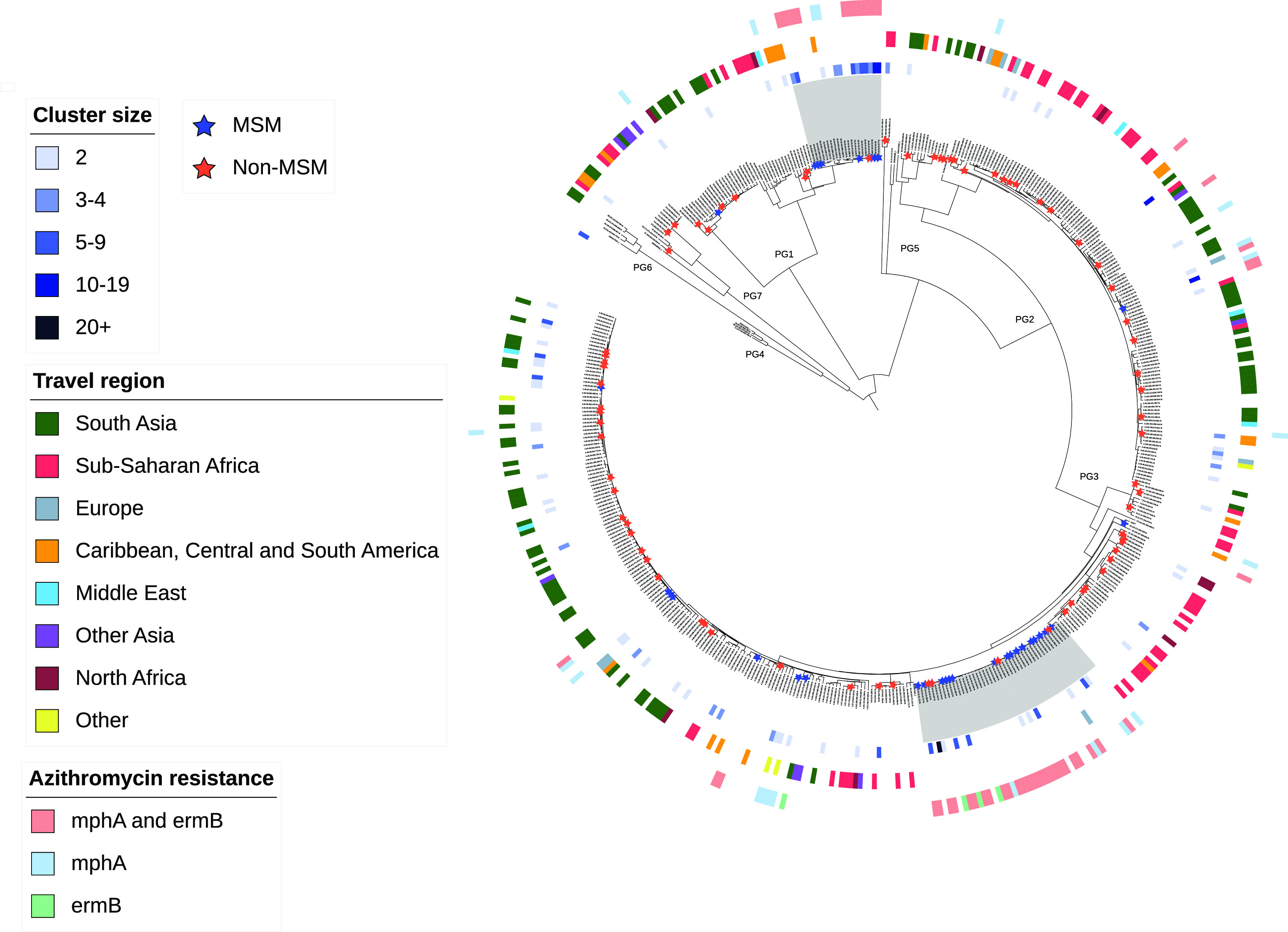
Phylogeny of S. flexneri CC245 isolates. Midpoint rooted maximum likelihood phylogenetic tree showing a single representative from each 10-SNP single linkage cluster (N = 474) for CC245 during the study period and seven reference strains for each phylogenetic group (27). The number of isolates represented by each tip (i.e., the number of cases within each 10-SNP single linkage cluster) ranges from 1 to 240. Clusters with two or more cases are shown as a colored track on the outside of the tree according to size range (inner track). Region of travel in the past 4 days (middle track) and genotypic markers of azithromycin resistance (outer track) are also shown as colored tracks on the outside of the tree. Isolates belonging to MSM and non-MSM (as reported in the questionnaire) are shown as stars on the branches, where each star indicates that at least one isolate from that cluster was MSM or non-MSM. Domestically circulating clades (referred to in the text as PG1 and PG3) are highlighted in gray: Top clade PG1 (serotype 3a) median pairwise SNP distance 37, minimum 0, maximum 165; bottom clade PG3 (serotype 2a) median pairwise SNP distance 21, minimum 0, maximum 47.

Phylogenetic analysis highlighted two domestically circulating clades first described by Baker et al. (2015, 2018) (5, 14) that were presumed to be associated with transmission through sex between men (Fig. 1); one phylogenetic clade within S. flexneri phylogenetic group 1 (PG1) serotype 3a and a second phylogenetic clade within PG3 serotype 2a (herein ‘PG1 and PG3’). These two clades accounted for 43.0% (398/926) of all isolates in this study. Where recorded, 97.5% (384/394) of PG1 and PG3 isolates were from adult men (≥18 years), 1.5% (6/394) were from adult women and 1.0% (4/394) were from children.

Among isolates from MSM (reported through the questionnaire), 88.4% (84/95) belonged to PG1 and PG3 (Fig. 1, Table 1). The remaining 11.6% (11/95) were dispersed throughout the phylogeny within lineages that were predominantly linked with travel to high-risk regions (here, “travel-associated lineages”). Six of 27 men self-identifying as heterosexual and seven of nine men not providing sexual identity or behavior information had isolates that were phylogenetically located within PG1 and PG3. Among the six heterosexual-identifying men, three reported recent sexual contact with a woman, one reported no recent sexual contact, and two provided no information.

**TABLE 1:** Selected epidemiological and molecular characteristics for S. flexneri cases with questionnaire data^*a*^

Characteristic	MSM N = 95	Other adults N = 61	Children N = 17	Not known N = 9
Sex				
Male Female	95 (100) 0	27 (44.3) 34 (55.7)	9 (52.9) 8 (47.1)	9 (100) 0
Age group				
<18 18−24 25−34 35−44 45−64 65+	0 8 (8.4) 33 (34.7) 25 (26.3) 28 (29.5) 1 (1.1)	0 6 (9.8) 24 (39.3) 8 (13.1) 17 (27.9) 6 (9.8)	17 (100) 0 0 0 0 0	0 1 (11.1) 0 3 (33.3) 5 (55.6) 0
Ethnic group				
White Asian or Asian British Other Not specified	70 (79.6) 3 (3.4) 15 (17.1) 7	22 (40.7) 18 (33.3) 14 (25.9) 7	2 (12.5) 11 (68.8) 3 (18.8) 1	5 (100) 0 0 4
Recent foreign travel history (past 4 days)^*b*^				
South Asia Sub-Saharan Africa Europe Caribbean, Central and South America Middle East North Africa Other Asia No/not specified^*b*^	0 0 10 (10.5) 1 (1.1) 1 (1.1) 0 0 83 (87.4)	18 (29.5) 17 (27.9) 1 (1.6) 5 (8.2) 2 (3.3) 3 (4.9) 1 (1.6) 14 (23.0)	9 (52.9) 2 (11.8) 1 (5.9) 0 1 (5.9) 1 (5.9) 0 3 (17.7)	0 0 1 (11.1) 0 1 (11.1) 0 0 7 (77.8)
Sexual identity (*n* = 165)				
Gay man Bisexual man Heterosexual man Heterosexual woman Not specified	92 (98.9) 1 (1.1) 0 0 2^*c*^	0 0 27 (50.0) 27 (50.0) 7^*d*^	- - - - -	0 0 0 0 9
Recent sexual contact (*n* = 131)				
Yes – with man Yes – with woman Yes – sex of partner not disclosed No Not specified	68 (72.3) 0 0 26 (27.7) 1	0 12 (50.0) 0 12 (50.0) 3	- - - - -	0 0 2 (33.3) 4 (66.7) 3
IMD quintile of deprivation				
1 (Most deprived) 2 3 4 5 (Least deprived) Not specified	40 (42.1) 38 (40.0) 9 (9.5) 3 (3.2) 5 (5.3) 0	20 (32.8) 20 (32.8) 14 (23.0) 4 (6.6) 3 (4.9) 0	5 (29.4) 8 (47.1) 2 (11.8) 2 (11.8) 0 0	1 (12.5) 6 (75.0) 0 1 (12.5) 0 1
Occupation				
School/nursery child Health care^*e*^ Social care/nursery worker^*e*^ Food handler/catering^*e*^ Fitness/gym worker Travel industry Other Not working/retired Not specified	0 8 (8.9) 3 (3.4) 8 (8.9) 0 2 (2.2) 55 (61.1) 14 (15.6) 5	0 1 (1.8) 4 (7.3) 6 (10.9) 2 (3.6) 0 34 (61.8) 8 (13.1) 6	17 (100) 0 0 0 0 0 0 0 0	0 1 (12.5) 0 0 0 0 6 (75.0) 1 (12.5) 1
Serotype				
2a Other Not specified	73 (86.9) 11 (13.1) 11	27 (45.8) 32 (54.2) 2	10 (62.5) 6 (37.5) 1	5 (55.6) 4 (44.4) 0
Phylogenetic lineage/clade				
PG3, serotype 2a PG1, serotype 3a Travel-associated lineage	71 (74.7) 13 (13.7) 11 (11.6)	5 (8.2) 1 (1.6) 55 (90.2)	0 0 17 (100)	5 (55.6) 2 (22.2) 2 (22.2)
Genotypic markers of azithromycin resistance				
*mphA* and *ermB* *mphA* only *ermB* only None	71 (74.7) 4 (4.2) 4 (4.2) 16 (16.8)	5 (8.2) 1 (1.6) 0 55 (90.2)	0 0 0 17 (100)	5 (55.6) 1 (11.1) 0 3 (33.3)
HIV status at *Shigella* diagnosis				
HIV diagnosed >6 wks previously HIV diagnosed within 6 wks HIV diagnosed >6 wks after Living with HIV but diagnosis date not known HIV negative/unknown	45 (47.9) 3 (3.2) 1 (1.1) 1 (1.1) 45 (47.9)	3 (4.9) 0 0 0 58 (95.1)	0 0 0 0 17 (100)	2 (22.2) 0 0 0 7 (77.8)

a N = 182 unless specified otherwise; denominator for sexual identity includes adults aged 18 years or older and the denominator for recent sexual contact (past 4 days prior to symptoms) includes adult men aged 18 years or older only. Missing data excluded from percentage calculations except for recent foreign travel history. IMD, Index of Multiple Deprivation; PG, phylogenetic group; MSM, men who have sex with men.

b Recent foreign travel (past 4 days prior to symptoms) as recorded on questionnaire; data missing for 1 case.

c Sexual identity not specified for two men, but recent same-sex sexual contact reported.

d Sexual identity not specified for 7 adult women.

e Occupation indicates the patient belongs to a recognized risk group and poses an increased risk of spreading their infection to others.

### Azithromycin resistance.

Overall, 40.0% (370/926) of isolates harbored *mphA* and/or *ermB* genes, known to confer azithromycin resistance. 89.2% (330/370) of these isolates fell phylogenetically within PG1 and PG3 and the remaining 10.8% (40/370) belonged to multiple other distinct phylogenetic branches (Fig. 1).

Among isolates with linked questionnaire data, 83.2% (79/95) of isolates from MSM carried *mphA* and/or *ermB* compared to only 7.7% (6/78) of isolates from non-MSM cases (*P* < 0.001) (Fig. 1, Table 1). The latter were from heterosexual-identifying men whose isolates fell phylogenetically within PG1 and PG3.

### Overlap between *Shigella* and HIV.

Among all S. flexneri isolates taken from PLWH; 98.3% (170/173) were from adult men and 1.7% (3/173) three were from adult women (Table 2). Among PLWH, 86.1% (149/173) had isolates that fell within PG1 and PG3. All these cases were adult men, and the probable route of HIV exposure was sex between men for 90.6% (135/149), heterosexual contact for 4.7% (7/149), injecting drug use for 1.3% (2/149) and unknown for 3.4% (5/149). Of all S. flexneri isolates within PG1 and PG3, 37.4% (149/398) were from PLWH.

**TABLE 2:** Epidemiological and molecular characteristics among S. flexneri cases living with HIV^*a*^

Characteristic	MSM clade (N = 149)	Travel-associated lineage (N = 24)
Sex		
Man Woman	149 (100) 0	21 (87.5) 3 (12.5)
Age group		
18−24 25−34 35−44 45−64	8 (5.4) 40 (26.9) 57 (38.3) 44 (29.5)	0 3 (12.5) 5 (20.8) 16 (66.7)
Recent foreign travel		
Yes No/not specified	7 (4.7) 142 (95.3)	7 (29.2) 17 (70.8)
Sexual identity (*n* = 55)		
Gay man Bisexual man Heterosexual man Not specified	44 (93.6) 1 (2.1) 2 (4.3) 2	5 (83.3) 0 1 (16.7) 0
Probable route of exposure to HIV		
Sex between men Injecting drug use Heterosexual contact – man Heterosexual contact – woman Not known	135 (93.8) 2 (1.4) 7 (4.9) 0 5	16 (76.2) 0 3 (14.3) 2 (9.5) 3
HIV status at time of S. flexneri diagnosis		
HIV diagnosed more than 6 wks previously HIV diagnosed within previous 6 wks HIV diagnosed within 6 wks after HIV diagnosed more than 6 wks after Living with HIV but diagnosis date not known	131 (88.5) 4 (2.7) 3 (2.0) 10 (6.8) 1	21 (87.5) 1 (4.2) 2 (8.3) 0 0
CD4 count (cells/mm^3^)		
≤350 >350 Not known	14 (16.7) 70 (83.3) 65	3 (20.0) 12 (80.0) 9
Viral load (c/ml)		
≤50 >50 Not known	74 (77.9) 21 (22.1) 54	13 (76.5) 4 (23.5) 7

a N = 173 unless otherwise specified. Sexual identity only available for people with a questionnaire (*n* = 55). Missing data excluded from percentage calculations, except for recent foreign travel. Recent foreign travel (past 4 days prior to symptom onset) as recorded on questionnaire or on laboratory request forms for people who did not have a questionnaire. MSM, men who have sex with men.

### Characteristics of cases with a questionnaire.

Among cases with questionnaire data, MSM were less likely to report recent foreign travel compared to non-MSM (12.6% [12/95] versus 78.2% [61/78], *P* < 0.001) (Table 1). Most MSM reporting recent foreign travel had visited Europe (83.3%, *n* = 10/12) whereas most non-MSM reporting recent foreign travel had visited other regions (98.4%, *n* = 60/61), predominantly South Asia (44.3%, *n* = 27/61) or sub-Saharan Africa (31.1%, *n* = 19/61). Most MSM reported recent sex with a same-sex partner (72.3%, *n* = 68/94), of whom 13.2% (9/68) had also traveled, mainly to Europe (*n* = 7). Among MSM without recent sexual contact (27.7% [26/94]), only two had recently traveled and this was to Europe. Most MSM were White (79.6% [70/94]), whereas 41.4% (29/70) of non-MSM were Asian ethnicity and 34.3% (24/70) were White (*P* < 0.001). Half (47/95) of MSM were living with diagnosed HIV at the time of S. flexneri diagnosis. Where available, 80.8% (21/26) had an undetectable viral load (≤50 c/ml) and 81.2% (18/21) had a CD4 count >350 cells/mm^3^.

### Novel strain transmission among MSM.

**FIG 2:**
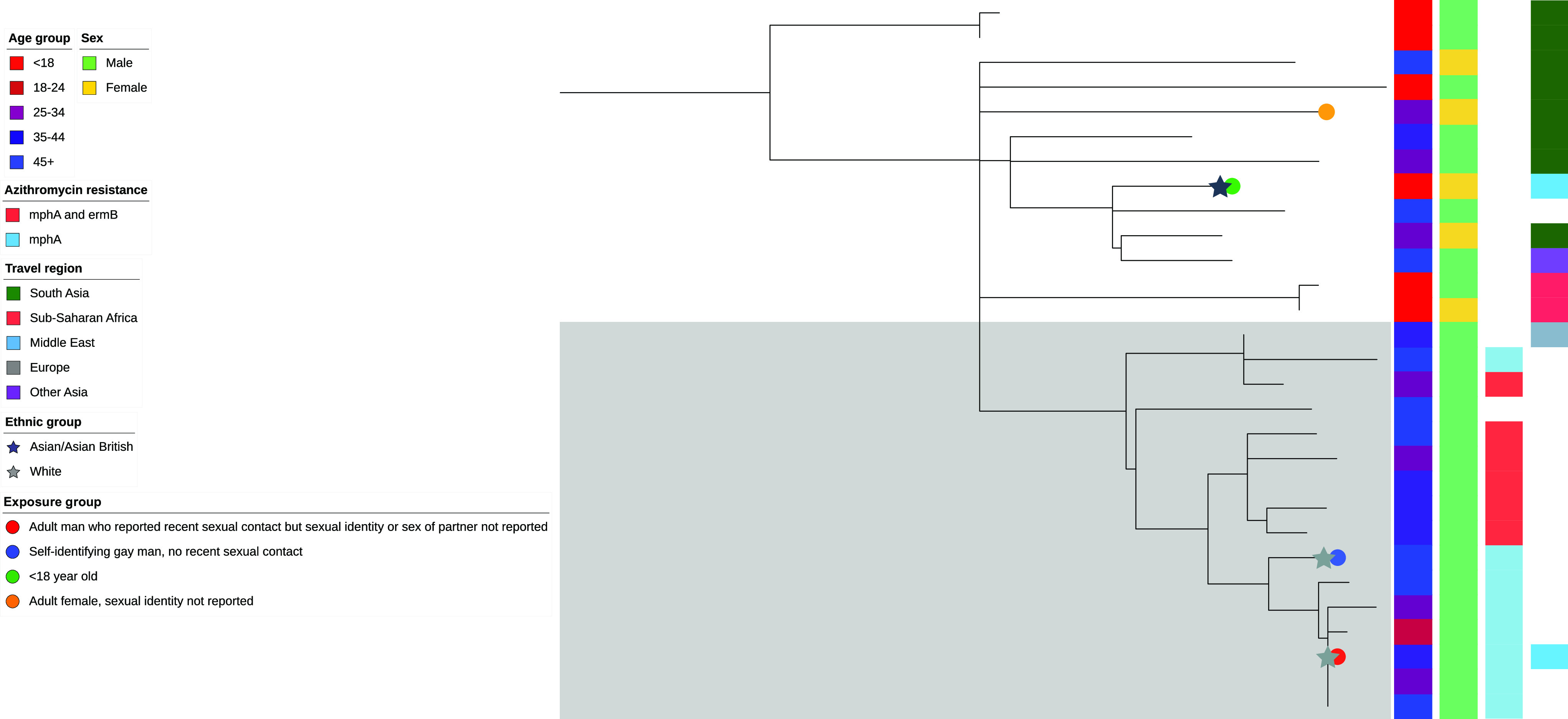
Detection of novel strain transmission among MSM. Midpoint rooted maximum likelihood phylogenetic tree of all isolates from one selected single linage cluster at the 50-SNP threshold nested within a travel-associated lineage (phylogenetic group 2, serotype 1c, SNP address 3.47.85.%, *n* = 29). Epidemiological data are represented as colored strips (age group, sex, genotypic markers of azithromycin resistance, region of travel) or as symbols on the branches (S. flexneri exposure group, ethnic group). Symbols are presented for people that have questionnaire data only. The clade associated with novel strain transmission in probable MSM is highlighted in gray (*n* = 16).

In addition to PG1 and PG3, the phylogenetic tree revealed new lineages suggestive of sexual transmission between men, most notably one exemplar cluster within PG2 (Table 3). Phylogenetic analysis of isolates within this cluster, contextualized using phylogenetically proximate isolates within 50 SNPs, revealed a previously unrecognized probable MSM clade (Fig. 2). All isolates within this clade were from adult men, most of whom had not traveled abroad (87.5%; 14/16), and most harbored genotypic markers of azithromycin resistance (87.5%; 14/16). Five were from men living with HIV, four of whom probably acquired HIV through sex between men. Questionnaire data were available for two adult men in this clade: One self-identified as gay and the other reported recent sexual contact but did not disclose their sexual identity or sex of their partner. In contrast, proximal isolates outside this clade were from a mixed group of adult men, women, and children, most of whom reported recent travel to a high-risk region (92.3%; 12/13), and none harbored genotypic markers of azithromycin resistance. Questionnaire data were available for an adult female and a child who had recently traveled to South Asia, and the Middle East, respectively.

**TABLE 3:** Epidemiological and molecular characteristics of 10-SNP clusters nested within travel-associated lineages and containing isolates from men who have sex with men^*a*^

10-SNP cluster	PG (serotype)	MSM cases	Non-MSM	Total cases in cluster	Males: Females in cluster	Foreign travel	*mphA* and/or *ermb*	Living with HIV^*b*^
78.324.644.966.1208.%	1 (3a)	1	0	2	2:0	0	1	1
3.47.85.237.397.%	2 (1c)	1	0	10	10:0	2^*c*^	10	3
4.49.69.337.364.%	3 (2a)	2	0	2	2:0	1	0	0
4.49.69.307.330.%	3 (2a)	1	0	2	2:0	0	2	2
4.49.69.95.615.%	3 (2a)	1	1	1	1:0	0	0	1
4.49.49.281.297.%	3 (2a)	1	1	2	2:0	0	0	0
4.110.188.317.439.%	3 (2a)	1	1	2	2:0	0	2	1
4.110.162.273.287.%	3 (2a)	1	1	2	2:0	0	2	1
4.184.304.462.531.%	3 (2a)	1	1	1	1:0	0	0	0
42.115.171.292.308.%	3 (2a)	1	1	1	1:0	0	1	1

a A single representative from each 10-SNP cluster was presented in Fig. 1. MSM cases (n = 11) as reported on questionnaire. Non-MSM cases (n = 6) as reported on questionnaire. Clusters with one case indicate that the isolate did not cluster with another isolate at the 10-SNP threshold. Exemplar cluster showing a strong signal of an unrecognized lineage that is being transmitted in probable MSM is highlighted in gray (3.47.85.237.397.%). PG, phylogenetic group; MSM, men who have sex with men.

b Where reported, probable exposure to HIV was reported as sex between men.

c Travel destination not recorded for one case.

### Clinical characteristics and outcomes.

Clinical characteristics for cases with questionnaire data are presented in Table 4. Among adults who reported receiving antimicrobials, the class was available for 59.3% (67/113). Most reported receiving ciprofloxacin alone (51.1% [22/43] for MSM, 76.2% [16/21] for other adults, and all three adults whose sexual identity and behavior were not reported). Nine MSM received more than one antimicrobial, of whom seven received ciprofloxacin in combination with at least one other antimicrobial, up to a maximum of three. Six MSM received azithromycin (azithromycin only [*n* = 3], azithromycin and doxycycline [*n* = 2], azithromycin and ciprofloxacin [*n* = 1]); all had isolates harboring genotypic markers of azithromycin resistance.

**TABLE 4:** Clinical characteristics, antimicrobial treatment, and health-seeking behavior among S. flexneri cases with questionnaire data^*a*^

	MSM N = 95	Other adults N = 61	Children N = 17	Not known^*b*^ N = 9
Previously heard of shigellosis^*c*^				
No Yes Not specified	55 (64.0) 31 (36.0) 9	50 (87.7) 7 (12.3) 4	13 (92.9) 1 (7.1) 3	5 (83.3) 1 (16.7) 3
Healthcare received				
GP Hospital Sexual Health/HIV clinic GP & Hospital GP, Hospital & Sexual Health/HIV clinic Not specified	32 (34.8) 28 (30.4) 13 (14.1) 12 (13.0) 7 (7.6) 3	36 (62.1) 9 (15.5) 1 (1.7) 12 (20.7) 0 (0) 3	6 (37.5) 3 (18.8) 0 (0) 7 (43.8) 0 (0) 1	4 (57.1) 2 (28.6) 1 (14.3) 0 (0) 0 (0) 2
Diarrhoea				
No Yes Not specified	1 (1.1) 92 (98.9) 2	1 (1.7) 59 (98.3) 1	0 16 (100) 1	0 9 (100) 0
Abdominal pain				
No Yes Not specified	6 (7.1) 78 (92.9) 11	6 (10.3) 52 (89.7) 3	4 (26.7) 11 (73.3) 15	1 (12.5) 7 (87.5) 1
Vomiting				
No Yes Not specified	54 (66.8) 27 (33.3) 14	36 (69.2) 16 (30.8) 9	6 (33.3) 10 (66.7) 2	4 (50.0) 4 (50.0) 1
Fever				
No Yes Not specified	17 (19.5) 80 (80.5) 8	20 (38.5) 32 (61.5) 9	3 (17.7) 14 (82.4) 0	2 (28.6) 5 (71.4) 2
Mucus in stools				
No Yes Not specified	31 (43.1) 41 (56.9) 23	34 (70.8) 14 (29.2) 13	5 (50.0) 5 (50.0) 7	3 (37.5) 5 (62.5) 1
Blood in stools				
No Yes Not specified	25 (29.4) 60 (70.6) 10	29 (52.7) 26 (47.3) 6	9 (69.2) 4 (30.8) 4	4 (50.0) 4 (50.0) 1
Admitted to hospital				
No Yes Not specified	55 (62.5) 33 (37.5) 7	46 (79.3) 12 (20.7) 3	9 (56.3) 7 (43.8) 1	6 (66.7) 3 (33.3) 0
Antimicrobials prescribed				
No Yes Not specified	18 (19.6) 74 (80.4) 3	25 (43.9) 32 (56.1) 4	0 (0) 16 (100.0) 1	1 (12.5) 7 (87.5) 1

a N = 182. Missing data excluded from percentage calculations. MSM, Men who have sex with men.

b Not known includes adult men who did not provide information on sexual identity or recent sexual behavior.

c Based on the question: Has the person heard of shigellosis/*shigella* spp. before?

Hospital admission was reported in 29.1% (48/165) of adults and was positively associated with: being MSM (aOR MSM versus non-MSM: 2.20 [95% CI: 1.02 to 4.73]; *P* = 0.038), S. flexneri serotype 2a (aOR all other serotypes versus serotype 2a: 0.28 [95% CI: 0.11 to 0.72], *P* = 0.004), and no recent foreign travel (aOR foreign travel versus no foreign travel: 0.34 [95% CI: 0.15 to 0.75]; *P* = 0.005) (Table 5). There was no evidence for an association between hospital admission and HIV status. Antimicrobial use was positively associated with: being MSM (aOR MSM versus non-MSM: 3.27 [95% CI: 1.62 to 6.63]; *P* < 0.001), S. flexneri belonging to PG1 or PG3 (aOR travel-associated lineage versus PG1/PG3: 0.46 [95% CI: 0.23 to 0.91]), S. flexneri harboring genotypic markers of azithromycin resistance (aOR: 2.82 [95% CI: 1.41 to 5.64]; *P* = 0.003), no recent foreign travel (aOR foreign travel versus no foreign travel: 0.35 [95% CI: 0.18 to 0.69]; *P* = 0.002), being of White ethnicity (aOR ethnic minority group versus White: 0.26 [95% CI: 0.12 to 0.54]; *P* < 0.001), and living with HIV (aOR: 2.15 [95% CI: 1.00 to 4.64]; *P* = 0.043).

**TABLE 5:** Characteristics associated with hospital admission and antimicrobial use in adults diagnosed with S. flexneri and with linked questionnaire data^*a*^

	Hospital admission			Antimicrobial use		
	n/N (%)	OR (95% CI)	aOR (95% CI)	n/N (%)	OR (95% CI)	aOR (95% CI)
Exposure group (N = 156)						
Non-MSM MSM *P* value	12/61 (19.7) 33/95 (34.7)	1.00 2.17 (1.02-4.65) 0.039	1.00 2.20 (1.02-4.73) 0.038	32/61 (52.5) 74/95 (77.9)	1.00 3.19 (1.59-6.42) 0.001	1.00 3.27 (1.62-6.63) <0.001
HIV status (N = 164)						
Negative/unknown Living with HIV *P* value	30/112 (26.8) 17/52 (32.7)	1,00 1.33 (0.65-2.71) 0.439	1.00 1.33 (0.65-2.71) 0.443	71/112 (63.4) 41/52 (78.9)	1.00 2.15 (1.00-4.64) 0.043	1.00 2.15 (1.00-4.65) 0.043
Serotype (N = 152)						
2a Other *P* value	36/105 (35.0) 6/47 (12.8)	1.00 0.28 (0.11−0.72) 0.004	1.00 0.28 (0.11−0.72) 0.004	72/105 (68.6) 30/47 (63.8)	1.00 0.81 (0.39-1.67) 0.567	1.00 0.81 (0.39-1.67) 0.567
Phylogenetic lineage/clade (N = 165)						
PG1/PG3 Travel-associated lineage *P* value	31/97 (32.0) 17/68 (25.0)	1.00 0.71 (0.35-1.42) 0.330	1.00 0.71 (0.35-1.45) 0.346	73/97 (75.3) 40/68 (58.8)	1.00 0.47 (0.24-0.92) 0.026	1.00 0.46 (0.23-0.91) 0.025
Azithromycin resistance (N = 165)					
No Yes *P* value	21/74 (28.4) 27/91 (29.7)	1.00 1.06 (0.54−2.09) 0.856	1.00 1.05 (0.52-2.09) 0.897	42/74 (56.8) 71/91 (78.0)	1.00 2.70 (1.38-5.32) 0.003	1.00 2.82 (1.41-5.64) 0.003
Foreign travel (N = 165)						
No/unknown Yes *P* value	38/104 (36.5) 10/61 (16.4)	1.00 0.34 (0.16−0.75) 0.005	1.00 0.34 (0.15-0.75) 0.005	80/104 (76.9) 33/61 (54.1)	1.00 0.35 (0.18-0.70) 0.003	1.00 0.35 (0.18-0.69) 0.002
Age group (N = 165)						
18−24 25−34 ≥35 *P* value Per yr (age as a continuous variable) *P* value (age as a continuous variable)	3/15 (20.0) 20/57 (35.1) 25/93 (26.9)	0.68 (0.18–2.61) 1.47 (0.72–3.00) 1.00 0.401 1.00 (0.97–1.02) 0.793	0.68 (0.18–2.61) 1.47 (0.72–3.00) 1.00 0.401 1.00 (0.97–1.02) 0.793	10/15 (66.7) 38/57 (66.7) 65/93 (69.9)	0.86 (0.27–2.75) 0.86 (0.42–1.75) 1.00 0.907 1.00 (0.97–1.02) 0.930	0.86 (0.27–2.75) 0.86 (0.42–1.75) 1.00 0.907 1.00 (0.97–1.02) 0.930
Ethnic group (N = 147)						
White Ethnic minorities *P* value	26/97 (26.8) 17/50 (34.0)	1.00 1.41 (0.67–2.94) 0.367	1.00 1.42 (0.68–2.97) 0.358	77/97 (79.4) 25/50 (50.0)	1.00 0.26 (0.12–0.55) <0.001	1.00 0.26 (0.12–0.54) <0.001
IMD quintile (N = 164)						
1−2 (Most deprived) 3 4–5 (Least deprived) *P* value	37/125 (29.6) 8/23 (34.8) 3/16 (18.8)	1.00 1.27 (0.50–3.24) 0.55 (1.15–2.04) 0.531	1.00 1.28 (0.50–3.32) 0.56 (0.15–2.08) 0.534	85/125 (68.0) 17/23 (73.9) 11/16 (68.8)	1.00 1.33 (0.49–3.64) 1.04 (0.34–3.18) 0.850	1.00 1.34 (0.48–3.68) 1.04 (0.33–3.21) 0.850

a Total numbers vary for each question due to missing data. Unadjusted and age-adjusted odds ratios (ORs) and 95% confidence intervals (CIs) calculated using logistic regression. Models adjusted for age as a continuous variable. P values by likelihood ratio test. Reference category for age group is aged 35 years and over. MSM, men who have sex with men; PG, phylogenetic group; IMD, Index of Multiple Deprivation.

## DISCUSSION

We describe the largest study of shigellosis in England and build on previous studies by providing more comprehensive data on sexual identity and behavior, and by describing and comparing clinical characteristics and outcomes in MSM and other exposure groups. By including all S. flexneri CC245 isolates that were referred to the reference laboratory over a 2-year period, our findings are generalizable nationally. We show that most S. flexneri isolates from MSM belonged to two domestically circulating clades (PG1 and PG3) and the majority harbored genotypic markers of azithromycin resistance. We also identified a new lineage associated with transmission through sex between men that was nested within travel-associated lineages, illustrating that shigellae have likely been introduced to the MSM population following recent travel to a high-risk region.

About one third of PG1 and PG3 isolates in our study were taken from PLWH, primarily acquired through sex between men, indicating the overlap between these epidemics. Of concern, MSM and PLWH were more likely than other cases to be treated with antimicrobials for their shigella infection, and MSM were also more likely to be hospitalized. The latter is consistent with a study from the USA that reported higher odds of severe shigellosis (hospitalisation, bacteremia or death associated with S. flexneri) in adult men compared to women, although sexual identity and behavior were not assessed (17).

HIV is a known risk factor for shigellosis (18, 19) and HIV-related immunosuppression has been associated with more severe illness prior to the introduction of highly active antiretroviral therapy (20–22). There were limited data in our study on CD4 count and HIV load, so we were unable to explore whether these factors were associated with shigellosis severity. Where data were available, most had an undetectable viral load and a high CD4 count. However, although the clinical implications are unclear, even with effective treatment there is evidence that gut mucosal immunity may not be fully restored (23, 24). The association between HIV status and antimicrobial use might also indicate more frequent health care attendance and thereby opportunities to collect stool specimens for microbiological investigations for PLWH. Clinicians might also be inclined to prescribe antimicrobials in PLWH to avoid further complications (25, 26).

We found a strong association between hospital admission and infection with S. flexneri serotype 2a that could reflect the presence of specific virulence determinants (27, 28). As MSM were more likely to be infected with S. flexneri serotype 2a than non-MSM, this could also partly explain more frequent hospital admissions among MSM.

Previous studies have found that MSM diagnosed with shigellosis are often coinfected with or have a recent history of bacterial STIs (7). Azithromycin has been used for the treatment of many STIs and it is likely that off-target exposure from STI treatment has favored selection of azithromycin resistance in MSM-associated strains of *Shigella* spp. Although azithromycin is not the primary treatment for shigellosis (5), some MSM infected with azithromycin-resistant isolates were prescribed azithromycin, either alone or in combination with other antimicrobials. The high frequency of genotypic markers of azithromycin resistance among MSM isolates is concerning and indicates azithromycin is unsuitable for treating shigellosis in MSM. Furthermore, it highlights the need for a holistic approach to antimicrobial stewardship in this population that considers the long-term consequences of frequent antimicrobial exposure and bystander selection of resistance.

Unsurprisingly, we found the main exposure of MSM was recent sex with a man and that of non-MSM was recent travel to a high-risk region. However, 28% of MSM reported no recent sexual contact, which could suggest nonsexual transmission, a delay in symptom onset (incubation period greater than 4 days), relapse of a previously acquired chronic infection, or misreporting. Of note, a small number of isolates from PG1 and PG3 were from women, children, heterosexual men and other adult men who did not provide sexual identity or behavior information. It is possible that some of the adult men in these clades had sex with other men but did not disclose this due to perceived stigma (29, 30). Nonetheless, while transmission networks may overlap, there was no evidence of sustained transmission between networks of MSM and the wider community in England.

Our study has limitations. Questionnaire data were only available for HPTs who participated in the pilot and these covered geographical regions with proportionally larger MSM populations (31), thereby favoring questionnaire responses from MSM. Some antimicrobial treatment may have been prescribed for a coinfection (e.g., azithromycin for a bacterial STI). Insufficient sample size and the high level of correlation between independent variables prevented a multivariable analysis of the characteristics associated with clinical severity controlling for confounding. We only included cases presenting to health care who had a specimen referred to the reference laboratory; a third of isolates cultured from stool samples at diagnostic hospital laboratories may not be referred and many less severe cases may remain undiagnosed (12). This under ascertainment of cases might have led to bias in our study because many factors are likely to influence whether an isolate is included, for instance health-seeking behavior and testing practices, which in turn may be influenced by severity of illness, age, the immune status of the individual or existing comorbidities (13, 32).

Our study illustrates the relevance and importance of sex between men as a route of transmission for S. flexneri, with multiple phylogenetically distinct lineages expanding clonally in MSM. Combining WGS, epidemiological and clinical data provides unique insights into S. flexneri transmission that can inform contact tracing, targeted prevention (e.g., washing hands and genitals before and after sex, using a barrier for risk practices and avoiding the use of shared sex toys [26, 33]) and appropriate referral for STI, HIV and blood-borne virus testing. We show that people will answer questions on sexual identity and behavior during public health follow-up and recommend they be included routinely in shigellosis investigations. Future studies should seek to explain why MSM experience more severe clinical outcomes. It is currently unclear whether this is related to the infecting pathogen, the gut microbiome or immune status of the individual, behavioral factors that influence the infectious dose, or a combination of these. Future studies would require a larger sample of cases to help distinguish the actual effect of each factor.

## MATERIALS AND METHODS

### Isolate collection.

The UK Standards for Microbiology Investigations request that all isolates of *Shigella* spp. from diagnostic hospital laboratories are submitted to the national reference laboratory for species identification and molecular typing. In this study, we included all S. flexneri isolates referred to the national reference laboratory by diagnostic hospital laboratories in England between August 2015 and July 2017. Demographic data (name, date of birth, sex, postcode and foreign travel history) were available from laboratory request forms. Full details of the isolates used in this study have been reported previously (6).

### Epidemiological and clinical data.

We used data from two sources: a standardized exposure questionnaire used for public health follow-up of shigellosis cases (Supplementary File 1) and information collected as part of routine national HIV surveillance (34).

In the UK, shigellosis is statutorily notifiable, and all suspected cases are routinely contacted by a local health protection team (HPT) at PHE as part of public health follow-up and case management. The shigellosis questionnaire was designed to standardize and expand the information collected during case follow-up and was piloted between August 2015 and March 2017 in seven of the 21 HPTs in England (all three in London and four outside London) (6). Information was collected on sexual identity (cases aged ≥18 years), recent sexual contact (men aged ≥18 years), foreign travel, and food and water consumption (all within 4 days of symptom onset), clinical condition (including hospital admission and antimicrobial treatment), and whether the case belonged to a recognized risk group for onward transmission (e.g., food handler, health care worker, or having contact with children aged ≤5 years [10]). Data linkage to laboratory isolates used name, date of birth, sex, and postcode (6).

New HIV diagnoses among people aged ≥15 years are routinely reported to PHE on a voluntary basis by laboratories and clinicians from a variety of National Health Service (NHS) settings in England, Wales, and Northern Ireland (35). These data are supplemented with epidemiological and follow-up clinical data reported by all HIV outpatient clinics to create a national cohort of PLWH, known as the HARS. Data on new HIV diagnoses and care of PLWH in Scotland are submitted to PHE by Public Health Scotland and integrated with the national data set. Before submitting data to PHE, reporters convert the patient’s surname to a soundex code (an anonymous identifier [36]). A hierarchical matching algorithm based on this soundex code, first initial, date of birth, postcode, and sex was used to link HARS to all S. flexneri isolates, including the subset with a questionnaire (18). HIV diagnosis date, probable route of exposure to HIV, most recent CD4 count and HIV load (within 3 months of S. flexneri diagnosis) were included in the final linked data set and all personal identifiers were removed.

### WGS and sequencing analysis.

Genomic DNA was extracted using the QiaSymphony DNA extraction platform (Qiagen) and WGS was performed using the Illumina HiSeq 2500 platform. Sequencing analysis was performed using a standardized pipeline (6, 37). Further details are provided in Supplementary File 2. Hierarchical single linkage clustering was performed on the pairwise single nucleotide polymorphism (SNP) distance matrix at descending distance thresholds (250, 100, 50, 25, 10, 5 and 0) (37). For phylogenetic analyses, recombinant regions of the genome were removed using Gubbins v2.0 (38) and RAxML v8.2.8 was used to create maximum-likelihood trees under the General Time Reversible model using up to 1000 bootstrap replicates (39). Tree annotation was performed using Interactive Tree Of Life (iTOL) v4.3 (40, 41).

### Data analysis.

We analyzed demographics and foreign travel history information alongside molecular data (phylogenetic inferences and genotypic markers of azithromycin resistance) for all S. flexneri cases included in the study. We also explored the characteristics of cases living with HIV. For cases with questionnaire data, characteristics were described for MSM (men who self-identified as gay or bisexual, or who reported recent same-sex sexual contact), and all other cases combined (heterosexual men, women, and children <18 years old, herein ‘non-MSM’). Differences between these two groups were assessed using the Chi-squared test (adult men who did not provide sexual identity or behavior information were excluded).

For S. flexneri cases with questionnaire data, we explored epidemiological, molecular and clinical characteristics associated with markers of clinical severity (25) using both univariable and age-adjusted logistic regression. These analyses were restricted to adult cases only. Characteristics associated with two different clinical outcomes were explored: hospital admission and antimicrobial use (as reported on the shigellosis questionnaire). Missing responses for these clinical outcomes were treated as absence of the specific outcome.

All analyses were restricted to isolates belonging to S. flexneri CC 245 as this was the dominant CC observed during the study period (92% of isolates) and all isolates from MSM belonged to CC245.

### Ethical considerations.

PHE has authority to collect and handle patient data for public health monitoring and infection control under Regulation 3 of the Health Service (Control of Patient Information) Regulations 2002. The PHE Caldicott Panel approved this analysis in June 2017. To ensure anonymity of PLWH, the final data set was irreversibly anonymised prior to analysis.

### Data availability.

FASTQ reads from all sequences can be found under the PHE Pathogens Bioproject (PRJNA315192) at the National Center for Biotechnology Information (NCBI) Read Archive: https://www.ncbi.nlm.nih.gov/bioproject/315192. The Short Read Archive accession numbers are available in the Supplementary Data File.
