# Supplementary material for: Linkage of Whole Genome Sequencing, Epidemiological, and Clinical Data to Understand the Genetic Diversity and Clinical Outcomes of Shigella flexneri among Men Who Have Sex with Men in England
Source: Microbiol Spectr. 2021 Dec 15;9(3):e01213-21. doi: 10.1128/Spectrum.01213-21 (PMC8672885; doi:10.1128/Spectrum.01213-21)
Supplement: SUPPLEMENTAL FILE 2 — Supplemental material. Download SPECTRUM01213-21_Supp_1_seq6.pdf, PDF file, 0.3 MB [file spectrum01213-21_supp_1_seq6.pdf]

Supplementary Material

Supplementary File 1. Standardised shigellosis exposure questionnaire

Standardised shigellosis exposure questionnaire used by Health Protection Teams (HPTs) participating in a national pilot

Shigella *flexneri/dysenteriae/boydii* questionnaire

1: QUESTIONNAIRE DETAILS

Interviewer name:		Interview date :	
Interviewer organisation:		Interviewer Telephone:	
Person interviewed name and relationship to case:			

2: CASE CLASSIFICATION AND ID

PHE reference no. e.g. HPZone					
Environmental health ID no.(if relevant)					
If relevant: GUM clinic name		GUM clinician		GUM clinic no.	
Local laboratory result: Culture					
PCR					
Local laboratory specimen number		Local Laboratory name			
Reference laboratory result					
		Confirmed	Probable	Possible	
Classification	*Shigella flexneri*	☐	☐	☐	
	Shigella dysenteriae	☐	☐	☐	
	Shigella boydii	☐	☐	☐	
	Shigella species	☐	☐	☐	

3: PERSONAL AND IDENTIFYING DETAILS

First name:		Family name/Surname:			
Address:					
Postcode:		Tel (h):		Tel (m):	
Email:					
Sex:	M ☐ F ☐	Date of birth (dd/mm/yyyy):		Age:	yrs
NHS No:		GP name:			
GP address:				GP Tel:	

Are there any children living in the household? (other than the case)	Y ☐	N ☐	How many? ☐

Public Health England is committed to ensuring all individuals are treated equally and fairly, and therefore we ask all people the following questions about their ethnicity and sexual orientation. This also helps us to identify and investigate outbreaks.

**Ethnicity:**				
White	☐ British	☐ Irish		☐ Other (please state)
Mixed	☐ White/Black Caribbean	☐ White/Black African	☐ White/Asian	☐ Other (please state)
Asian/Asian British	☐ Indian	☐ Pakistani	☐ Bangladeshi	☐ Other (please state)
Black/Black British	☐ Caribbean	☐ African		☐ Other (please state)
Chinese	☐ Chinese			
Other	☐ (please state)			

**Sexual identity (if over 18 years old)**
I will now read out a list of terms people sometimes use to describe how they think of themselves.
☐ Heterosexual or straight
☐ Gay or lesbian
☐ Bisexual
☐ Other
As I read the list again please say ‘yes’ when you hear the option that best describes how you think of yourself.(Pause briefly after each option during second reading).
☐ Spontaneous don’t know/refusal

4: RISK GROUPS

Occupation			
Work/School/Nursery name:		Tel:	
Contact person			
Address:			
Postcode:		Date last attended:	

Does the patient fit into any of the following categories (tick all that apply)?:		
**Group A**	☐	Any person of doubtful personal hygiene or with unsatisfactory toilet, hand washing or hand drying facilities at home, work or school. Particular consideration should be given as to whether individual infant-school-aged children (aged 6 or 7 years) are able to satisfactorily observe good personal hygiene. Health protection personnel (LA and HPU) should agree locally on how to make this assessment in engagement with parents or teachers/carers.
**Group B**	☐	All children aged five years old or under including those who attend school, pre-school, nursery or other childcare or minding groups.
**Group C**	☐	People whose work involves preparing or serving unwrapped food to be served raw or not subjected to further heating.
**Group D**	☐	Clinical, social care or nursery staff who work with young children, the elderly, or other particularly vulnerable people, and whose activities increase the risk of transferring infection via the faecal-oral route. Such activities include helping with feeding or handling objects that could be transferred to the mouth
**No risk group**	☐	

5: CLINICAL DETAILS

Onset date:		Still ill:		If no, →	Duration of illness (days): .
Symptoms:	Diarrhoea Yes ☐ No ☐		Vomiting Yes ☐ No ☐		
	Fever Yes ☐ No ☐		Blood in stools Yes ☐ No ☐		
	Abdominal pain Yes ☐ No ☐		Mucus in stool Yes ☐ No ☐		
Healthcare sought from:	☐ GP visit	☐ A&E	☐ Sexual health clinic	☐ Other (specify):	
Date of stool sample:					
Admitted to hospital for this illness:	Yes ☐ No ☐		Admission date:		
Hospital name:			Duration of stay (d):		
Treated with antibiotics:	Yes ☐ No ☐		If Y, specify:		
Has the patient heard of *Shigella* before?	Yes ☐ No ☐				

6: TRAVEL

6.1 In the **FOUR** *(boydii, flexneri)* or **SEVEN** (*dysenteriae)* days prior to illness, did you arrive or return to the UK from **ABROAD**?	Yes ☐ No ☐

Specify countries visited (from most recent)

**Country/Region**	**Date arrived**	**Date departed**	**Details**

6.1 In the **FOUR** *(boydii, flexneri)* or **SEVEN** (*dysenteriae)* days prior to illness, did you travel elsewhere **WITHIN** the UK?

**Town/Resort**	**Date arrived**	**Date departed**	**Details**

7: OTHER RISK FACTORS

7.1 In the **FOUR** *(boydii, flexneri)* or **SEVEN** (*dysenteriae)* days prior to illness, did you have any contact with:

Anyone with diarrhoea?	Yes ☐ No ☐	If yes, details:	
Children under 5 years?	Yes ☐ No ☐	If yes, details:	
Visitors from UK or overseas?	Yes ☐ No ☐	If yes, details:	
Attend a gym/ swimming pool/other communal sports facilities?	Yes ☐ No ☐	If yes, where?	

8: SEXUAL CONTACT (ONLY for MALE cases aged18 years or older. If under 18 years please go straight to section 9). Please ask about sexual contact regardless of travel history or other identified risk factors.

8.1 We ask all adult men with Shigella about sexual contact as Shigella can be sexually transmitted via the faecal-oral route. Men who have sex with men (MSM) are at greater risk of acquiring the infection than others and there have been outbreaks of shigella among the MSM population.

Did you have sexual contact with anyone in the **FOUR** (*boydii, flexneri)* or **SEVEN** (*dysenteriae)* days prior to illness			Yes ☐ No ☐
**IF** yes, was this with a	Male ☐ and/or	Female ☐	Prefer not to answer ☐

8.2 Public Health Advice: If the case is a MSM - please remember to provide them information on how to reduce transmission as per PHE leaflet (link below) and recommend they attend their local sexual health clinic for STI screening.

https://www.gov.uk/government/uploads/system/uploads/attachment_data/file/323532/Shigella_leaflet.pdf

If the case has been abroad to areas with a high risk for shigella infection in the FOUR *(boydii, flexneri)* or SEVEN (*dysenteriae)* days prior to illness, AND/OR sexual transmission has been identified as a likely source, please go straight to section 10.

High risk area - South America, Asia (including the Middle East) and Africa: Low risk areas - Europe, North America and Australia:

9: FOOD AND WATER HISTORY

9.1 Food prepared at home

Please list all food eaten in the **FOUR** *(boydii, flexneri)* or **SEVEN** (*dysenteriae)* before you became unwell. Describe what was eaten and when in the appropriate section on the chart. If possible give details of the shop where it was bought. **Routine cooking kills *Shigella* bacteria so particular attention should be given to raw and uncooked food, as well as baby foods.**

Days pre-onset	Date	Breakfast	Lunch	Evening meal	Snacks
1					
2					
3					
4					
5					
6					
7					

9.2 In the **FOUR** *(boydii, flexneri)* or **SEVEN** (*dysenteriae)* days before you became unwell, did you eat any food that was not prepared at home, either in this country or abroad (e.g. hotels, restaurants, cafes, pubs; school and work canteens; takeaways, fast food outlets; barbecues and picnics; social events; other people's homes)? If yes, enter details below

Date	Description of food	Establishment where food obtained

9.3 In the **FOUR** *(boydii, flexneri)* or **SEVEN** (*dysenteriae)* days before you became unwell, what was the source of your drinking water? (tick all that apply)

Mains ☐	Private ☐	Bottled ☐
Filtered ☐	Well ☐	Spring ☐
Other (specify) ☐		

10: PLEASE COMPLETE CONTACT SHEET ON THE FOLLOWING PAGE

11: FURTHER INFORMATION

Is this case part of an outbreak?	Yes ☐ No ☐	**Setting**
Are there any other possible exposures to infection not already discussed		
May we contact you again if we need to ask any further questions?	Yes ☐	No ☐

Please remember for provide public health advice to reduce risk of ongoing transmission

CONTACTS

PHE (HPZ) REF NUMBER: CASE NAME:

***Household/significant contacts: List everyone who lives in the same accommodation or shares the same kitchen, bathroom or toilet**.
^Type of symptom: D= Diarrhoea; F= Fever; Ab= Abdo pain; V= Vomiting; M= mucus in stool; and BS= Bloody stool.

	Name	Address	Date of birth	Relatio-nship to case*	Gen-der (M/F)	Sympt oms (Y/N)	Type of Symptom	Date of Onset	Risk Group (A/B/C/D /No)	GP Details	Exclusio n req'd (Y/N)	Contact advised re. excl-usion (Y/N)
1												
2												
3												
4												
5												

	Name	Address	Date of birth	Relatio-nship to case*	Gen-der (M/F)	Sympt oms (Y/N)	Type of Symptom	Date of Onset	Risk Group (A/B/C/D /No)	GP Details	Exclusio n req'd (Y/N)	Contact advised re. excl-usion (Y/N)
6												
7												
8												

Significant contact is defined as household/workplace/school contact and/or those who have been exposed to the similar circumstances as the case i.e. travelled with the case.

Supplementary File 2. Whole genome sequencing and sequencing analysis methods

Microbiological typing, including confirmation of the species, serotyping and single nucleotide polymorphism (SNP) analysis was performed at Public Health England (PHE) using whole genome sequencing (WGS) [1-3].

Genomic DNA from bacterial isolates was extracted using the QiaSymphony DNA extraction platform (Qiagen). DNA was fragmented and tagged for multiplexing with Nextera XT DNA Sample Preparation Kits (Illumina) and sequenced using the Illumina HiSeq 2500 platform at PHE. Bases with a Phred score below 30 (error probability of 1 in 1000) were removed from the trailing ends using Trimmomatic [4].

Species confirmation was performed by comparing kmers (short strings of DNA of length *k*; in this method, *k*=18) within the reads to a set of kmers found in a collection reference genomes [5]. The closest percentage match was identified and provided initial confirmation of the species. The kmer ID software is available here: https://github.com/phe-bioinformatics/kmerid.

Serotyping was based on the structure of the O-antigen using 'GeneFinder' (https://github.com/phe-bioinformatics/gene_finder) [6]. This customised algorithm utilises Bowtie2 alignment software [7] and SAMtools [8] to map newly sequenced reads to a database of sequences encoding the O-antigen synthesis and modification genes [2]. Only predictions of serotype that matched to a reference gene sequence at >80% nucleotide identity over >80% of length were accepted [2].

Sequence type (ST) assignment was performed by aligning the newly sequenced reads to a Multilocus Sequence Typing (MLST) database of reference alleles using Bowtie2 alignment software [7], as described by Tewolde *et al*. [9]. The software is available here: https://github.com/phe-bioinformatics/MOST).

For SNP typing analysis, reads were mapped to the reference strain (*S. flexneri* serotype 2a strain 2457T (Genbank accession: AE014073.1) using Burrows Wheeler Alignment-Maximal Exact Match (BWA-MEM).[10] The resulting Sequence Alignment Maps (SAM) were sorted and indexed to produce Binary Alignment Maps (BAM) using SAMtools [8]. High quality variant positions (Mapping Quality > 30, Depth > 10, Variant Ratio > 0.9) identified using GATK2 in unified genotyper mode [11] were extracted and stored in SnapperDB [12]. Hierarchical single linkage clustering was performed on the pairwise SNP distance matrix at descending SNP thresholds (250, 100, 50, 25, 10, 5 and 0) as previously

described [12]. The clustering is summarised as a ‘SNP address’ (a seven-digit code) which describes the cluster membership at each of the thresholds. For phylogenetic analyses, recombinant regions of the genome were identified using Gubbins and masked [13]. Pseudosequences of polymorphic positions were used to create a maximum likelihood tree using RAxML under the General Time Reversible model using up to 1000 bootstrap replicates [14]. Tree annotation was performed using Interactive tree of life (iTOL) v4.3 [15, 16].

Antimicrobial-resistance determinants were detected using ‘GeneFinder’ (https://github.com/phe-bioinformatics/gene_finder) [6]. Bowtie2 [7] was used to map newly sequenced reads to a database of reference sequences, followed by SAMtools to create BAM files [8]. Genes were defined as present if they represented 100% of the reference sequence, with greater than 90% nucleotide identity.

Supplementary File 3. Summary of *S. flexneri* isolates and linked data

Flow chart showing the total number of *S. flexneri* Clonal Complex (CC) 245 isolates referred to the national reference laboratory between August 2015 and July 2017, the number with linked questionnaire data and the number linked to the HIV and AIDS Reporting System (HARS). The national shigellosis exposure questionnaire was piloted between August 2015 and March 2017 by seven of 21 Health Protection Teams (HPTs).

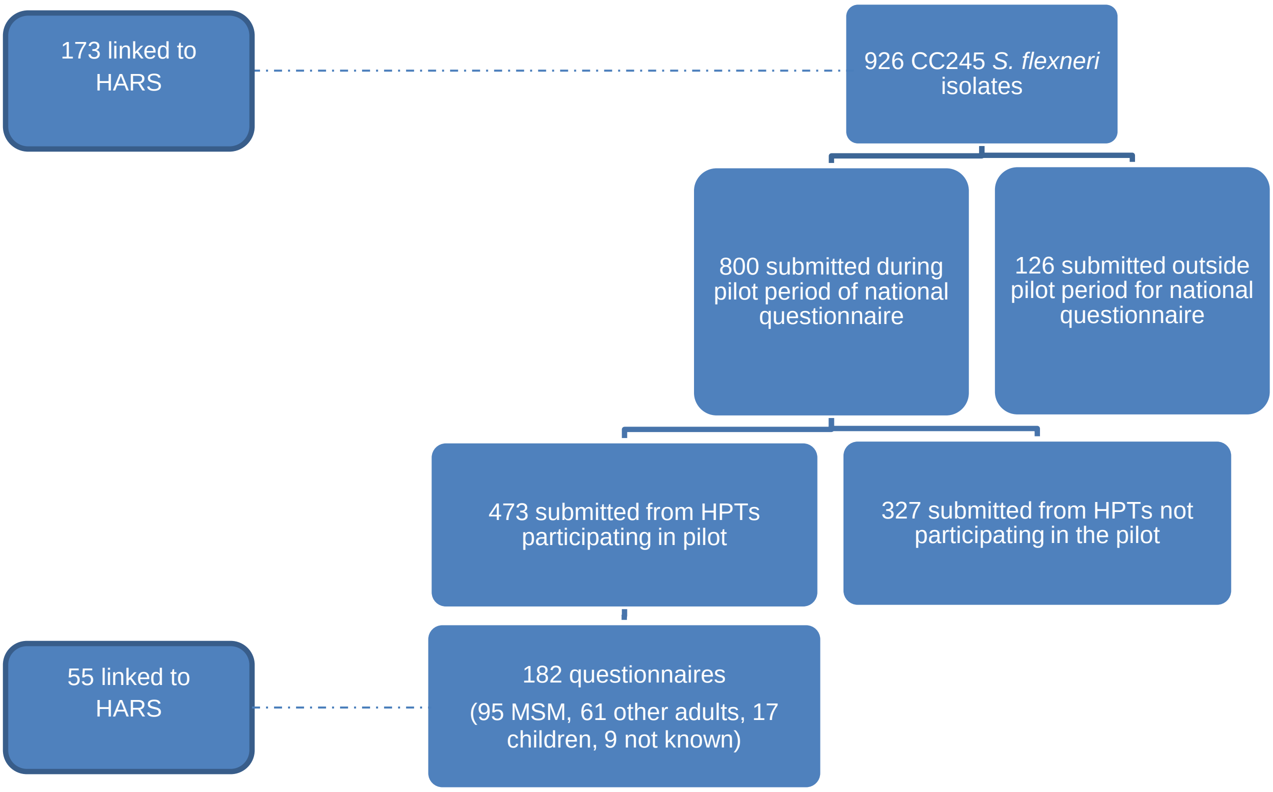

Supplementary File 4. Characteristics of *S. flexneri* isolates with and without linked questionnaire data in England, August 2015 to July 2017

Variable	Questionnaire (n=182)		No questionnaire: pilot HPTs (N=291)		p-value (questionnaire vs no questionnaire pilot HPTs)	No questionnaire: all isolates (n=744)		p-value (questionnaire vs no questionnaire all isolates)
	N	%	N	%		N	%	
**Health Protection Team (HPT)**								
London	137	75.3	186	63.9	0.010	245	32.9	<0.001
Outside London	45	24.7	105	36.1		499	67.1	
**Sex**								
Male	140	76.9	217	75.9	0.795	519	71.0	0.111
Female	42	23.1	69	24.1		212	29.0	
Not known	0	0	5			13		
**Age group**								
<18	17	9.3	39	13.5	0.492	128	17.3	0.016
18-24	15	8.2	25	8.6		67	9.1	
25-34	57	31.3	75	25.9		159	21.5	
35-44	36	19.8	66	22.8		141	19.1	
45+	57	31.3	85	29.3		243	32.9	
Not known	0	0	1			6		
**Recent foreign travel***								
No/unknown	146	80.2	226	77.7	0.509	547	73.5	0.062
Yes	36	19.8	65	22.3		197	26.5	

N=926 isolates. N=473 isolates submitted by Health Protection Teams (HPTs) participating in the pilot (August 2015 to March 2017).

*Recent foreign travel based on data reported on laboratory request forms only to enable comparison between two groups.

P-values calculated using Chi-squared test.

References

1. Bardsley M, Jenkins C, Mitchell HD, et al. Persistent Transmission of Shigellosis in England Is Associated with a Recently Emerged Multidrug-Resistant Strain of Shigella sonnei. J Clin Microbiol **2020**; 58(4).

2. Chattaway MA, Greig DR, Gentle A, Hartman HB, Dallman TJ, Jenkins C. Whole-Genome Sequencing for National Surveillance of *Shigella flexneri*. Front Microbiol **2017**; 8: 1700.

3. Mitchell HD, Mikhail AFW, Painset A, et al. Use of whole-genome sequencing to identify clusters of Shigella flexneri associated with sexual transmission in men who have sex with men in England: a validation study using linked behavioural data. Microb Genom **2019**; 5(11).

4. Bolger AM, Lohse M, Usadel B. Trimmomatic: a flexible trimmer for Illumina sequence data. Bioinformatics **2014**; 30(15): 2114-20.

5. Chattaway MA, Schaefer U, Tewolde R, Dallman TJ, Jenkins C. Identification of Escherichia coli and Shigella Species from Whole-Genome Sequences. J Clin Microbiol **2017**; 55(2): 616-23.

6. Doumith M, Godbole G, Ashton P, et al. Detection of the plasmid-mediated mcr-1 gene conferring colistin resistance in human and food isolates of Salmonella enterica and Escherichia coli in England and Wales. J Antimicrob Chemother **2016**; 71(8): 2300-5.

7. Langmead B, Salzberg SL. Fast gapped-read alignment with Bowtie 2. Nat Methods **2012**; 9(4): 357-9.

8. Li H, Handsaker B, Wysoker A, et al. The Sequence Alignment/Map format and SAMtools. Bioinformatics **2009**; 25(16): 2078-9.

9. Tewolde R, Dallman T, Schaefer U, et al. MOST: a modified MLST typing tool based on short read sequencing. PeerJ **2016**; 4: e2308.

10. Li H, Durbin R. Fast and accurate long-read alignment with Burrows-Wheeler transform. Bioinformatics **2010**; 26(5): 589-95.

11. McKenna A, Hanna M, Banks E, et al. The Genome Analysis Toolkit: a MapReduce framework for analyzing next-generation DNA sequencing data. Genome Res **2010**; 20(9): 1297-303.

12. Dallman T, Ashton P, Schafer U, et al. SnapperDB: a database solution for routine sequencing analysis of bacterial isolates. Bioinformatics **2018**; 34(17): 3028-9.

13. Croucher NJ, Page AJ, Connor TR, et al. Rapid phylogenetic analysis of large samples of recombinant bacterial whole genome sequences using Gubbins. Nucleic Acids Res **2015**; 43(3): e15.

14. Stamatakis A. RAxML version 8: a tool for phylogenetic analysis and post-analysis of large phylogenies. Bioinformatics **2014**; 30(9): 1312-3.

15. Letunic I, Bork P. Interactive tree of life (iTOL) v3: an online tool for the display and annotation of phylogenetic and other trees. Nucleic Acids Res **2016**; 44(W1): W242-5.

16. Letunic I, Bork P. Interactive Tree Of Life (iTOL) v4: recent updates and new developments. Nucleic Acids Res **2019**.
